# Draft Genome Sequence of Filamentous Marine Cyanobacterium Lyngbya confervoides Strain BDU141951

**DOI:** 10.1128/genomeA.00066-15

**Published:** 2015-03-05

**Authors:** Mathu Malar Chandrababunaidu, Diya Sen, Sucheta Tripathy

**Affiliations:** Structural Biology and Bioinformatics Division, CSIR, Indian Institute of Chemical Biology, Kolkata, West Bengal, India

## Abstract

Lyngbya confervoides strain BDU141951 is a fast-growing, unicellular, marine, nonheterocystous cyanobacterium forming long unbranched filaments inside sheaths. Here, we report the draft genome assembly of Lyngbya confervoides BDU141951 for the first time. The genome size is 8,799,693 bp and has 6,093 putative protein-coding genes assembled into 298 scaffolds.

## GENOME ANNOUNCEMENT

Cyanobacteria are well known for their ability to produce sustainable bioenergy (1). Many efforts are on to reengineer these organisms for overproduction of biofuels from fast-growing strains. Lyngbya fits very well into this category with its fast growth and stress tolerance, as it can grow in saline as well as in freshwater conditions. Several strains of L. aestuarii are capable of producing hydrogen gas (2), and some strains of L. aestuarii are also known to produce various antifungal compounds (3). Another species, L. officinalis, produces a toxin called Lyngbya toxin-A, which is characterized by its antiangiogenic and anti proliferative properties (4).

Lyngbya confervoides is a marine cyanobacterium that was cultured in ASN medium at room temperature (approximately 27°C) with 16-h light and 8-h dark periods. Cultures were gently shaken once a day. Morphologically, L. confervoides forms green clumps in liquid culture and is thick and matty in solid media. Genomic DNA was isolated using a Uniflex Bacterial DNA isolation kit (Genei, USA). The total amount of DNA isolated was approximately 3.6 μg, with a concentration of 330 ng/µl. Two libraries, one paired-end (300-bp insert size) and one mate-pair library (3-kb insert size), were constructed. Whole-genome sequencing was carried out using the Illumina HiSeq platform. Paired-end libraries were sequenced at 98× coverage (12.3 million reads), and the mate-pair library was sequenced at 40× coverage (5.6 million reads); the average read length was 101 bp for both libraries. Initially, the sequence artifacts and adapters were removed by using SGA (5) and Tagdust (6). The clean reads from both libraries were assembled using Allpaths LG-49856 (7). The final assembly was 8.7 Mb in length, having 298 scaffolds and an *N*_50_ of 5,207,129. The largest scaffold was 5,207,129 bp and the smallest scaffold was 3,620 bp in length. The calculated G+C content of the genome was 55%. The sequences were submitted to GenBank and were annotated using the PGAP pipeline (http://www.ncbi.nlm.nih.gov/genome/annotation_prok). There were 6,093 protein-coding genes, 1,096 pseudogenes, 4 CRISPR arrays, 70 tRNA genes, and 2 ncRNA genes predicted from this assembly. The protein sequences were submitted for KEGG analysis using the KAAS pipeline (8). Pathway analysis revealed a nonribosomal peptide synthetic pathway leading to the production of viomycin (Vioj), which has antituberculosis activity. Since Lyngbya survives higher salinity conditions, several salt-tolerant genes, such as spermidine synthase spermidine transporters (9), are found in the genomes. Spermidine synthase protein from Lyngbya confervoides BDU141951 shares closest similarity with Leptolyngbya sp. PCC 6406 at 83% identity. Genome mining of this organism will help find many important genes for metabolite production as well as stress tolerance for biotechnology applications.

### Nucleotide sequence accession number.

The Lyngbya confervoides BDU141951 draft genome sequence data have been deposited in GenBank under the accession number JTHE00000000.
